# Taurine reduces the secretion of apolipoprotein B100 and lipids in HepG2 cells

**DOI:** 10.1186/1476-511X-7-38

**Published:** 2008-10-17

**Authors:** Teruyoshi Yanagita, Seo-Young Han, Ying Hu, Koji Nagao, Hideaki Kitajima, Shigeru Murakami

**Affiliations:** 1 Department of Applied Biochemistry and Food Science, Saga University, Saga 840-8502, Japan; 2 Jeonnam Natural Resources Research Institute, 756 Kisanri Anyangmyeon Jangheung-gun Jeollanamdo 529-851, Korea; 3 R&D Headquarters, Self Medication Business, Taisho Pharmaceutical Co., Ltd., Tokyo 170-8633, Japan

## Abstract

**Background:**

Higher concentrations of serum lipids and apolipoprotein B100 (apoB) are major individual risk factors of atherosclerosis and coronary heart disease. Therefore ameliorative effects of food components against the diseases are being paid attention in the affluent countries. The present study was undertaken to investigate the effect of taurine on apoB secretion and lipid metabolism in human liver model HepG2 cells.

**Results:**

The results demonstrated that an addition of taurine to the culture media reduces triacylglycerol (TG)-mass in the cells and the medium. Similarly, cellular cholesterol-mass was decreased. Taurine inhibited the incorporation of [^14^C] oleate into cellular and medium TG, suggesting the inhibition of TG synthesis. In addition, taurine reduced the synthesis of cellular cholesterol ester and its secretion, suggesting the inhibition of acyl-coenzyme A:cholesterol acyltransferase activity. Furthermore, taurine reduced the secretion of apoB, which is a major protein component of very low-density lipoprotein.

**Conclusion:**

This is a first report to demonstrate that taurine inhibits the secretion of apoB from HepG2 cells.

## Background

Taurine, *β*-sulphonic amino acid was first isolated more than 150 years ago from ox bile [1]. Taurine is considered to be an essential nutrient for human infants and cats and distributed extensively in mammalian cells and tissues [1,2]. Its recognized metabolic function in liver is conjugation with bile acids, which is important for bile secretion and lipid digestion [3]. Nevertheless, taurine also has beneficial effects on the liver that include prevention and treatment of cholestasis and prevention of liver damaged due to toxic chemicals [4-6].

In past decades, much attention has been paid to the effects of taurine on bile acid metabolism. Conversely, a few studies concerning the role of taurine on the other liver lipids have been reported. Liver is the central organ of lipid metabolism. Previous in vitro studies with human hepatoblastoma cells showed that cellular levels of taurine were associated with the rate of bile acid synthesis, the reduction of free cellular cholesterol concentration and the higher expression of low density lipoprotein (LDL) receptor activity [7]. Similarly, in vivo studies have shown that supplementation with taurine influences the serum cholesterol levels in the experimental animals [8]. This was associated with the alterations of activities of 7 *α*-hydroxylase and 3-hydroxy-3-methylglutarylutaryl coenzyme A reductase in the liver [9].

Thus, it appears that taurine involves not only in biliary lipid secretion, but also in cholesterol metabolism in the liver [10]. This possibility is also supported by the previous reports showing taurine-related changes in liver lipid composition of the experimental animals [11,12].

However, even though liver is the central organ of lipid metabolism, only a few studies concerning the effect of taurine on liver lipid metabolism has been reported. In addition, the mechanism for these effects has been not worked out in detail. In addition, there are controversies about the effect of taurine on serum lipid concentrations in human beings [1,13].

Taurine biosynthesis derives from the trans-sulphuration pathway originating with methionine [14]. Liver and serum taurine levels in rats are reported to be approximately 3.62 μmol/g liver, and 2.5 μmol/ml serum, respectively [15].

The liver is the organ to synthesize and secrete the lipoproteins containing apolipoprotein B100 (apoB). ApoB is an essential structural component of very low density lipoprotein (VLDL) and LDL and is required for the intracellular assembly and the secretion of these lipoproteins [16,17]. The biological role of VLDL is primarily related to TG transport and eventually to cholesterol transport to the tissues. The synthesis of all these components occur on endoplasmic reticulum (ER) membranes and nascent VLDL particles are assembled from these components before the lipoprotein formation and secretion is accomplished. As the elevation of the concentration of apoB as well as of LDL cholesterol is regarded as an important risk factor for coronary artery disease. It is important to investigate the factors controlling the secretion rate of apoB-containing lipoprotein by the liver.

The present studies were designed to examine whether taurine alters apoB secretion and lipid metabolism using the human hepatoblastoma cells, HepG 2, as a model of human liver. HepG2 cells have been found to retain many typical functions of the normal human hepatocytes, including lipoprotein and apolipoprotein synthesis [17-19].

## Results

### Protein content, 3-(4,5-dimethylthiazol-2-yl)-2,5-diphenyl tetrazolium bromide (MTT) cytotoxicactivity and taurine concentration in the cells

**Figure 1:**
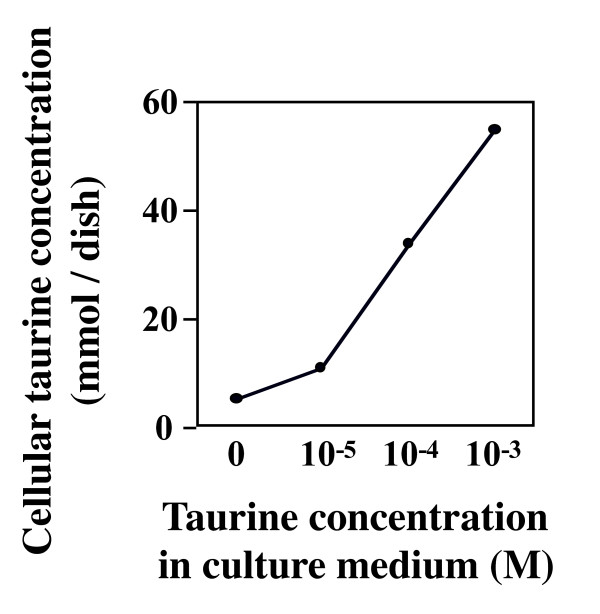
**HepG2 cells were cultured in the DMEM with or without taurine for 24 hr (n = 3).** Taurine content was measured by using HPLC method.

As shown in Figure 1, the concentration of cellular taurine was increased in a dose-dependent manner by increasing concentration of taurine added to the medium. No significant changes were noted in the cellular protein content by an addition of 10^-5^, 10^-4^, and 10^-3 ^M of taurine under the present culture medium. Taurine did not affect MTT activity, suggesting that taurine has no any cytotoxic effect under the present experimental conditions.

### Effect of taurine on triacylglycerol and cholesterol contents in the cells and the medium

**Figure 2:**
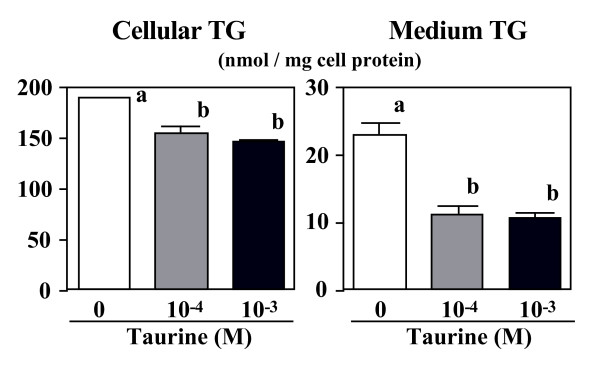
**Effect of taurine on TAG-mass in the cells and in the medium.** HepG2 cells were cultured in the DMEM with (10^-4 ^or 10^-3 ^M) or without taurine for 24 hr. Values are expressed as mean ± SE (n = 4). ^a, b ^Different letters show significant differences at *P *< 0.05.

**Figure 3:**
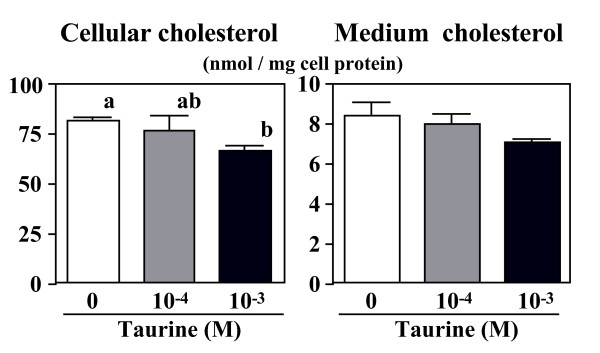
**Effect of taurine on cholesterol-mass in the cells and in the medium.** HepG2 cells were cultured in the DMEM with (10^-4 ^or 10^-3 ^M) or without taurine for 24 hr. Values are expressed as mean ± SE (n = 4). ^a, b ^Different letters show significant differences at *P *< 0.05.

The contents of TG and cholesterol in the cells and the medium were measured. When the cells were precultured in oleate-medium, taurine reduced cellular TG-mass significantly, and reduced the secreted TG (Figure 2). Similarly, cellular cholesterol-mass declined by up to 19% by taurine (Figure 3).

### Effect of taurine on lipid syntheses in the cells

**Figure 4:**
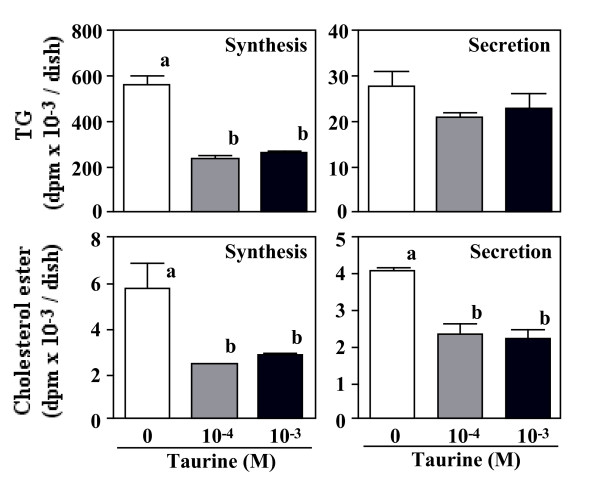
**Effect of taurine on the incorporation of [^14^C] oleic acid into cellular TAG and cholesterol-ester and their secretion.** HepG2 cells were cultured in the DMEM with (10^-4 ^or 10^-3 ^M) or without taurine for 24 hr. Values are expressed as mean ± SE (n = 4). ^a, b ^Different letters show significant differences at *P *< 0.05.

Figure 4 shows [^14^C] oleic acid uptake by TG and cholesterol ester in the cells and the medium. Taurine inhibited by up to 50% the incorporation of [^14^C] oleic acid into cellular TG. In addition, taurine reduced the synthesis of cholesterol ester from [^14^C] oleic acid by 58% in the cells and reduced its secretion into medium by up to 43%.

### Effect of taurine on ApoB secretion to the medium

**Figure 5:**
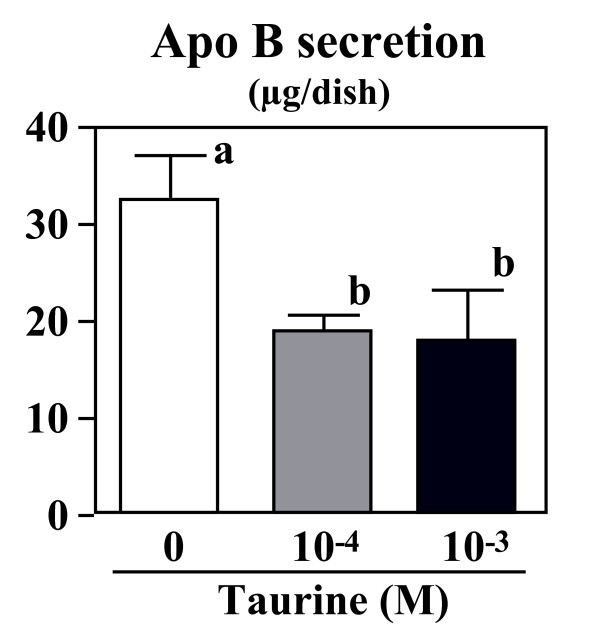
**Effect of taurine on apoB secretion into the medium.** HepG2 cells were cultured in the DMEM with (10^-4 ^or 10^-3 ^M) or without taurine for 24 hr. The apoB concentration in the medium was measured by using a sandwich enzyme-linked immunosorbent assay. Values are expressed as mean ± SE (n = 4). ^a, b ^Different letters show significant differences at *P *< 0.05.

Figure 5 shows the effect of taurine on apoB concentrations in the medium after 24 h incubation. Taurine at the concentrations of 10^-4 ^and 10^-3 ^M reduced apoB secretion by 42% and 45%, respectively.

## Discussion

ApoB-containing lipoproteins are assembled in the endoplasmic reticulum (ER), with maturation occurring in Golgi apparatus of hepatocytes prior to secretion [20]. ApoB is required for the assembly and secretion of apoB-containing lipoproteins which transport hydrophobic lipids, cholesterol ester, and TG in their cores. Regulation of the assembly and secretion of apoB-containing lipoproteins has become an active area of investigation as it is recognized that overproduction of apoB-containing lipoproteins may be responsible for coronary artery disease and hyperlipidemia.

The present study focused on examining the effects of taurine on the secretion of TG, cholesterol and apoB from HepG2 cells. Taurine had no affect on MTT activity and cellular protein content in the present cultured condition. Thus, it suggests that an addition of taurine from the concentrations of 10^-5 ^to 10^-3 ^M in the culture medium did not show any cytotoxic effect. When HepG2 cells contains a higher concentration of cellular TG by preincubated with oleic acid-rich medium, taurine remarkably reduced TG-mass (Figure 2) in both the cells and the medium. In the same condition, taurine reduced the incorporation of [^14^C] oleic acid into cellular TG (Figure 1), suggesting the inhibition of TG synthesis.

Taurine influenced cholesterol metabolism in HepG2 cells. In condition 1, taurine (10^-3 ^M) significantly reduced not only the cellular cholesterol-mass (Figure 3) but also the synthesis and secretion of [^14^C] oleoylcholesterol (Figure 4), suggesting the inhibition of cholesterol ester synthesis in the cells. It is well known that cholesterol ester formation is catalyzed by acyl-coenzyme A: cholesterol acyltransferase (ACAT) form free cholesterol and acyl-CoA. Reduction of the synthesis of cholesterol ester may suggest that relatively high amount taurine exerts for ACAT inhibitor.

ApoB is a major protein component of plasma VLDL and LDL. First, the effects of the concentration in the medium on apoB accumulation at the end of the incubation period were determined. We found that taurine inhibits apoB secretion from human hepatocytes preincubated with oleate medium (Figure 5). To determine if changes in intracellular lipid metabolism were responsible for the alteration on apoB secretion, appropriate radiolabeled tracer was added to the medium, the synthesis of cholesterol, cholesterol ester and TG was determined. Because high concentration of apoB has been implicated as risk factor for the development of atherosclerosis, reduction of apoB suggests the beneficial effect of taurine. Results suggest that there is an associated decrease in apoB secretion with a decrease in TG synthesis. Thus, a reduction of TG synthesis may inhibit VLDL-apoB secretion. An important factor determining VLDL-apoB secretion is the size of the intracellular TG pool [20]. Availability of TG for lipoprotein assembly greatly influences the size of VLDL particle secreted by the liver.

There is evidence to suggest the cholesterol ester can also regulate apoB secretion. Our group [19] and Dashti [21] reported that cholesterol ester synthesis might be a critical element for regulating apoB secretion by hepatocytes. Elevation of the serum cholesterol concentration is known to increase the risk of coronary heart disease. Depressed cholesterol ester formation by taurine may be beneficial for the prevention of such diseases. Recently microsomal triacylglycerol transfer protein (MTP) has been identified as a necessary factor for the assembly of TG-rich lipoproteins [22,23]. MTP is localized in the lumen of the endoplasmic reticulum in the liver and intestine, where it transfers TG, cholesterol ester, and phospholipids between phospholipid membranes [22,23]. However, effect of taurine on MTP activity remains to be determined.

## Conclusion

Overall, the present study demonstrated that taurine inhibits the secretion of cholesterol ester, TG and apoB in HepG2 cells. The reduction of lipid secretion is partly associated with the inhibition of cellular lipid synthesis. This is the first direct demonstration that taurine inhibits the secretion of apoB in HepG2 cells.

## Methods

### HepG2 cells culture

HepG2 cells were grown in DMEM supplemented with 10% FCS, penicillin (100 μg/ml), and streptomycin (100 μg/ml) at 37°C in an atmosphere containing 95% air and 5% CO_2 _[19,20]. The medium was renewed at intervals of 2–3 days. For experiments, a dish 3.5 cm in diameter and a 24-well plate were used and cells were plated out at a density of 100 × 10^4 ^cells/dish or 20 × 10^4 ^cells/well. Before the experiment were performed, cells were preincubated in 1% BSA-DMEM containing 1 mM oleic acid (oleate-medium) for 24 h, then cultured in 1% BSA-DMEM with or without (control) taurine (final concentration: 10^-5^, 10^-4^, and 10^-3 ^M) for 24 h (n = 3–4). MTT cytotoxicity [24] and protein concentration were determined.

### Determination of taurine concentrations in the cells

100 μl of cells and 400 μl of HClO_4 _(0.2 M) were placed into a microtube. After cautious shaking, the mixture was centrifuged (10000 rpm, 3 min, 4°C), then 100 μl of the supernatant was added to 350 μl of methanol and centrifuged again (10000 rpm, furan: methanol: sodium acetate = 2: 38: 360 (by volume). Taurine concentration was determined by HPLC according to the procedure.

### Determination of lipid content and cellular protein

The cellular protein concentration was determined using Lowry's method [25]. Total lipids in the cells and the medium were extracted and purified using the method of Bligh and Dyer [26]. The contents of TG and cholesterol were determined enzymatically using commercial kits (Wako Pure Chemical Industries, Co., Ltd., Osaka).

### Determination of lipids synthesis and their secretion

To assess the effects of the addition of taurine on the synthesis and secretion of TG and cholesterol ester in HepG2 cells of experiment 1, 0.5 μCi of [^14^C] oleic acid was added to each well. The incorporation of radioactivity into lipids and their secretion were measured after 24 h incubation. Total lipid was extracted as described above. Neutral lipid subclasses were separated by thin-layer chromatography precoated silica gel G with the solution mixtures petroleum ether: diethylether: acetic acid (80:20:1, by vol.) [27]. The radioactivity was measured with an imaging plate and BAS 2000 (Fuji Photo Film Co. Ltd. Kanagawa). The radioactivity was also measured by liquid scintillation counter (Wallic system 1410, Pharmacia, Uppsala, Sweden). Correction was made for background radioactivity and for quenching by the silica gel.

### Determination of apoB secretion into the medium of HepG2 cells

ApoB was measured by a sandwich enzyme-linked immunosorbent assay [28].

### Statistical analysis

Statistical analysis of data was performed by one-way ANOVA following by Fisher's PLSD test to establish differences among the groups. Differences were considered significant at P < 0.05.

## List of abbreviations

ACAT: acyl-coenzyme A cholesterol acyltransferase; ApoB: apolipoprotein B100; ER: endoplasmic reticulum; LDL: low-density lipoprotein; MTP: microsomal triacylglycerol transfer protein; MTT: 3-(4,5-dimethylthiazol-2-yl)-2,5-diphenyl tetrazolium bromide; TG: triacylglycerol; VLDL: very low-density lipoprotein.

## Competing interests

The authors declare that they have no competing interests.

## Authors' contributions

TY contributed in planning, analysis and publication of results. SYH contributed in experimental work and analysis. YH contributed in experimental work and analysis. KN contributed in the planning of the experiment and in discussion of results. JF contributed in planning of the experiment and in discussion of results. HK contributed in planning of the experiment and in discussion of results. SM contributed in planning of the experiment, discussion of results, and providing funding for the study.
